# Improved production of sublancin via introduction of three characteristic promoters into operon clusters responsible for this novel distinct glycopeptide biosynthesis

**DOI:** 10.1186/s12934-015-0201-0

**Published:** 2015-02-12

**Authors:** Shengyue Ji, Weili Li, Abdul Rasheed Baloch, Meng Wang, Binyun Cao

**Affiliations:** College of Animal Science and Technology, Northwest A&F University, 22 Xinong Road, Yangling, 712100 Shaanxi, P.R. China; College of Veterinary Medicine, Northwest A&F University, 22 Xinong Road, Yangling, 712100 Shaanxi, P.R. China

**Keywords:** Sublancin, Glycopeptide, Recombinant *B. subtilis* 1A747, Improved production, Transcriptional regulatory circuit

## Abstract

**Background:**

Sublancin is a novel and distinct antimicrobial glycopeptide that can be used as an alternative to conventional antibiotics. The reported production of sublancin by *Bacillus subtilis* 168 is poor because transcriptional regulatory circuit of *sunA*, a gene that encodes presublancin, is complex and difficult to control.

**Results:**

A strong inducible and easy to control vegetative σ^A^ promoter of P_*glv*_ was introduced to replace that of *sunA in situ* in *B. subtilis* 1A747 [SPβc, prototroph, the derivative of *B. subtilis* 168 (trpC2)]. Meanwhile, other two strong promoters of P43 and P_*luxS*_ were respectively placed before *sunI* and *sunT*–*bdbA*–*sunS*–*bdbB*, encoding five functional proteins that involved in the biosynthesis of mature sublancin. 642 mg sublancin was obtained from 1 L culture supernatant of recombinant *B. subtilis* 1A747 strains. Analysises of electrospray ionization mass spectrometry and circular dichroism spectrum showed that mature sublancin had a molecular weight of 3877.642 Da and displayed a α–helical conformation that are consistent with reported results. In addition, the mature sublancin was proved to be a potent antimicrobial glycopeptide with broad activity spectrum, moderate cytotoxicity and good conditional stability under high temperature, extreme pH and protease–rich environments, thus showing its potential for clinical applications.

**Conclusions:**

Our present findings suggest that recombinant *B. subtilis* 1A747 strains can effectively and efficiently biosynthesize mature sublancin. The replacement of native promoters provides an extra method for production improvement of some other complicated peptides such as nisin and subtilin.

**Electronic supplementary material:**

The online version of this article (doi:10.1186/s12934-015-0201-0) contains supplementary material, which is available to authorized users.

## Background

Natural peptides with post–translational modification are rapidly expanding class of agents with diverse biological activities [1]. Sublancin (Genbank accession number P68577.1) is a novel distinct peptide that is synthesized by *Bacillus subtilis* 168. This peptide can effectively kill specific pathogenic bacteria such as *Staphylococcus aureus* and *Streptococcus pyogenes* [2]. Sublancin is encoded by SPβ prophage in strains that lysogenize the SPβ bacteriophage and inhibits the growth of non–lysogenic strains [3]. Similar to lantibiotics [4], sublancin is firstly synthesized as a precursor with a double–glycine leader peptide in N–terminal and a core peptide in C–terminal, and the latter was post–translationally modified into mature peptide. However, unlike lantibiotics, sublancin has a unique post–translational S–glucosylation modification and is therefore considered as a distinct glycopeptide [5].

**Figure 1:**
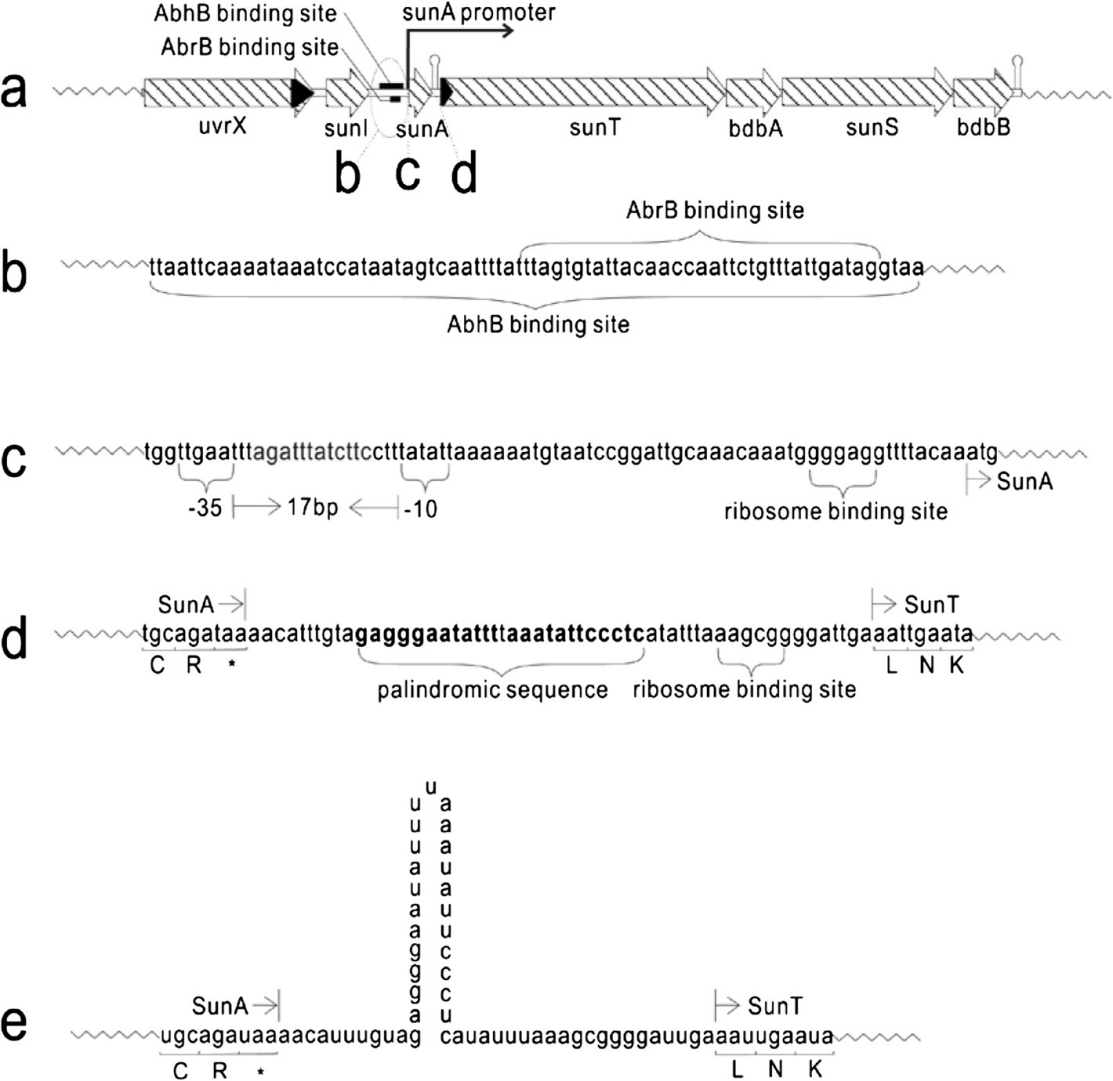
**Schematic of the sublancin gene cluster. (a)** The gene cluster of sublancin in *B. subtilis* 168 consists of the immunity protein gene *sunI*, precursor gene *sunA*, ABC–transporter gene *sunT*, two thiol–disulfide oxidoreductase genes *bdbA* and *bdbB*, and glycosyltransferase gene *sunS*. **(b)** Abr and AbhB binding sites are located upstream of the promoter of *sunA*. **(c)** A typical σ^A^ promoter is located upstream of *sunA*. **(d)** A palindromic sequence and a ribosome–binding site without promoter are located between *sunA* and *sunT*. **(e)** A hairpin structure is formed from the palindromic sequence after transcription.

The DNA fragment responsible for biosynthesizing mature sublancin is located in the prophage SPβ genome and includes two adjacent transcriptional units (*sunI* and *sunA*–*sunT*–*bdbA*–*sunS*–*bdbB*) with a length of 4.5 kb (Figure 1a). *sunI* provides the genetic basis for sublancin producer immunity [6]. Adjacently, an operon with five successive genes, *sunA*–*sunT*–*bdbA*–*sunS*–*bdbB*, is located immediately downstream of sunI. However, a terminator structure is present between *sunA* and *sunT* that, in some events such as sporulation happens in the DSM media, causes premature termination of the transcript (Figure 1a and e). The whole transcript of *sunA*–*sunT*–*bdbA*–*sunS*–*bdbB* is transcribed when mature sublancin is biosynthesized [7,8]. *sunA* is immediately located downstream of *sunI* and encodes the presublancin [2], and SunT is an ABC–type transporter with a proteolytic domain that removes the leader peptide from sublancin during its translocation across the membrane [9,10]. BdbA still remains unclear in sublancin biosynthesis although it has been presumed to have thiol oxidase activity [11]. BdbB belongs to the thiol–disulfide oxidoreductases and involved in the post–translational modification of disulfide bond formation in sublancin [9,11,12]. SunS is a S–glycosyltransferase that has a CxxS motif; and is involved in biosynthesis of mature sublancin by glucosylating Cys^22^ [13]. S–glycoside moiety of sublancin is important for conferring the antimicrobial activity [5].

Sublancin biosynthesis is controlled by a complex regulatory network that involves a minimum of five transcriptional regulators, namely Abh, AbrB, Rok, YvrG and YvrH (Additional file 1) [7,14,15]. Abh and AbrB directly bind to the overlapping regions within *sunA* regulatory region (Figure 1b). Abh plays a positive role in regulating the transition–stage *sunA* expression during vegetative growth. AbrB is a paralog of Abh that can transcriptionally repress the biosynthesis of sublancin [14,16]. The *in vitro* studies have shown that Rok can bind to *sunA* transcriptional regulatory region and its deletion improves the transcription of *sunA* and *sunT* [15]. YvrG and YvrH comprise of a novel two–component system, and simultaneously positively regulate the transcriptional units of *sunA* and *sunT*–*bdbA*–*sunS*–*bdbB* [7].

ECF σ factors belong to a subfamily of sigma 70 class and respond to various extracellular changes [17], and the regulation of antibiotic resistance functions is commonly mediated by these factors. *B. subtilis* harbors a minimum of seven known ECF σ factors *viz*., σ^M^, σ^W^, σ^X^, σ^Y^, σ^Z^, σ^V^, and σ^YlaC^ [18]. Of them, two ECF σ factors σ^M^ and σ^X^ with overlapping promoter specificity are involved in the biosynthesis–regulating of sublancin in *B. subtilis* 168 cells (Additional file 1) [19]. The monocistron *sunA*–*sunT*–*bdbA*–*sunS*–*bdbB* with a hairpin structure in between *sunA* and *sunT* (Figure 1d and e) is transcriptionally controlled under a σ^A^ promoter (Figure1a and c), and its typical motifs of −35 and −10 region are TTGACA and TATAAT with a consensus spacing of 17 nucleotides.

Natural production of sublancin biosynthesized by *B. subtilis* 168 is poor owing to its complex transcriptional regulatory mechanism [2]. Comparing to single polypeptide consisting of common amino acids [20], the mature sublancin undergoes further post–translational modification, including formation of the characteristic glucosylation moiety and disulfide bridges. It is not suitable for commercial production through conventional recombinant DNA technology or common peptide chemosynthesis method [1,2,5]. Considering the possibility of displacing the complex transcriptional regulatory mechanism to efficiently biosynthesize sublancin, we altered the transcriptional regulatory network of *sunA in situ* with a strong inducible P_*glv*_ vegetative σ^A^ promoter [21]. Meanwhile, other two strong promoters of P43 [22] and P_*luxS*_ [23] were placed before *sunI* and *sunT*–*bdbA*–*sunS*–*bdbB*, respectively.

## Results and discussion

### Construction of recombinant *B. subtilis* 1A747 strain

**Figure 2:**
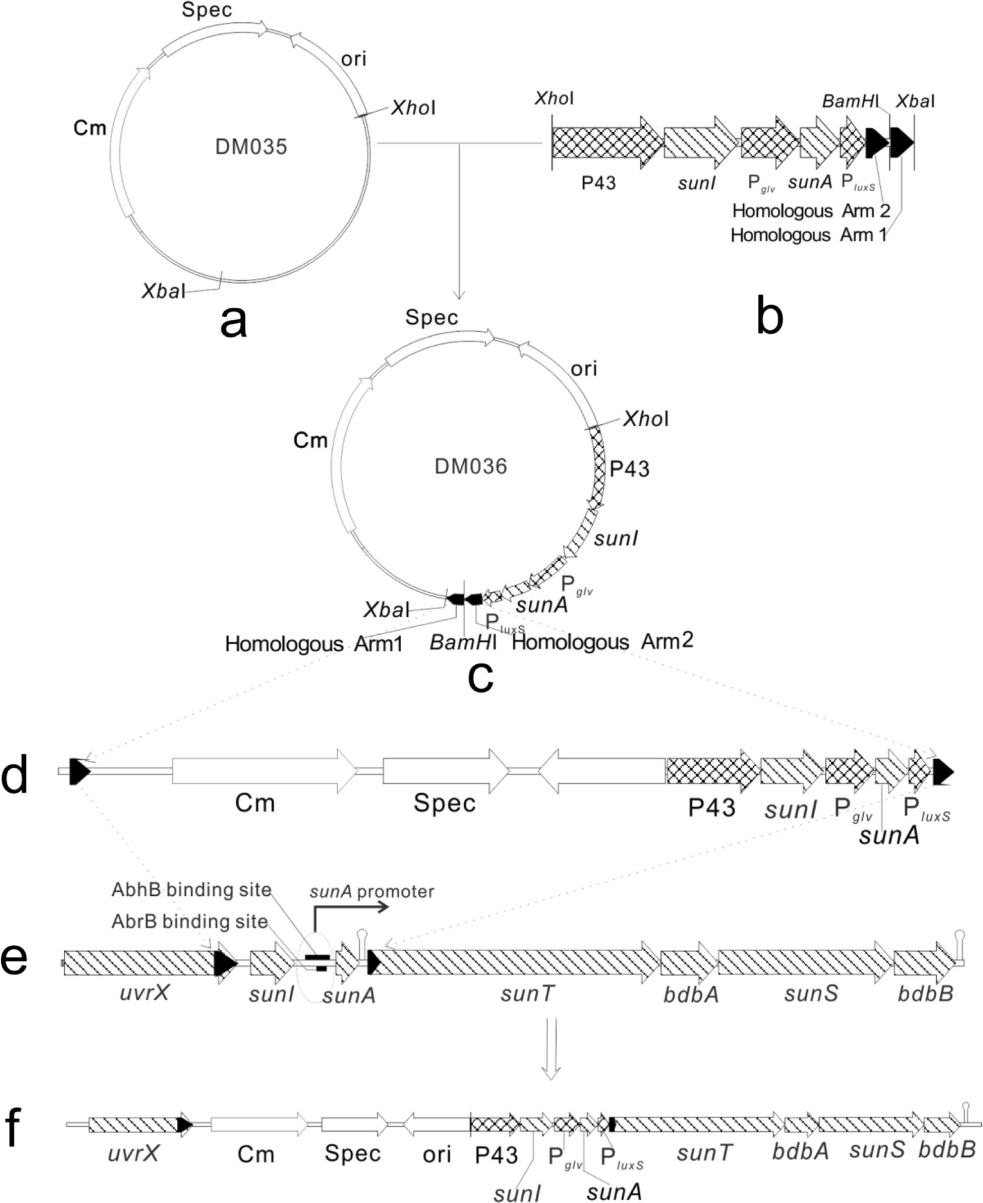
**Construction of recombinant** ***B. subtilis*** **1A747 strain. (a)** The plasmid pDM035 containing resistance genes of chloramphenicol and spectinomycin were double digested with *Xho*I and *Xba*I. **(b)** The fusion fragment of the P43–*sunI*–P_*glv*_–*sunA*–P_*luxS*_–two homologous arms with *Xho*I and *Sac*I was inserted into the vector pDM035 by displacing β–galactosidase gene to yield pDM036 **(c)**. **(d)** The pDM036 was digested and linearized with *BamH*I. **(e)** The linearized pDM036 was inserted into the prophage SPβ genome of *B. subtilis* 1A747. **(f)** The recombinant *B. subtilis* 1A747 strain was generated.

Three strong characteristic promoters including vegetative–and–stationary double functional promoter P43 [22], the maltose–inducible promoter P_*glv*_ [21] and vegetative promoter P_*luxS*_ [23], were *in situ* chromosome–integrated into *B. subtilis* 1A747 and respectively placed before two genes of *sunI* and *sunA* and one gene cluster of *sunT*–*bdbA*–*sunS*–*bdbB* that are responsible for mature sublancin biosynthesis. Then, recombinant *B. subtilis* 1A747 strains were constructed for the efficient biosynthesis of sublancin for commercial applications (Figure 2).

### Contribution of P43, P_*glv*_, and P_*luxS*_ on over–transcription of relative genes

**Figure 3:**
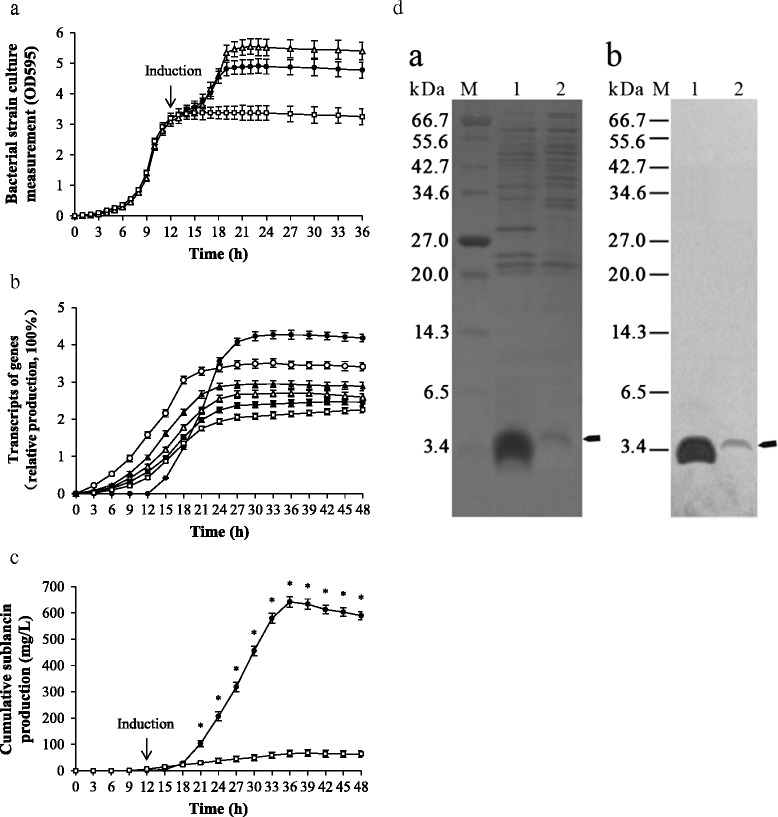
**Improved biosynthesis of sublancin in** ***B. subtilis*** **1A747 [SPβc, prototroph, derivative of** ***B. subtilis*** **168 (trpC2)] based on three strong heterologous promoters. (a)** The absorbances of *B. subtilis* cell cultures at OD595 of, harbouring P_*glv*_, P43, and P_luxS_, induced by maltose (●) and not induced by maltose (□), and not harbouring those three promoters also treated by maltose (∆). **(b)** Real–time PCR analysis of the transcription amounts of *sunA* (●), *sunI* (○), *sunT* (▲), *bdbA* (∆), *sunS* (■), and *bdbB* (□) of *B. subtilis* 1A747 harbouring P_*glv*_, P43, and P_luxS_ induced by maltose, compared with the control harbouring native promoters also treated with maltose. **(c)** Cumulative sublancin production in *B. subtilis* culture supernatant harbouring those three strong promoters induced by maltose (●) and not harbouring those promoters also induced by maltose (□), the maximum production of 628 mg sublancin was obtained from 1 L recombinant bacteria culture supernatant at 36 h after fermentation. **(d)** Tricine–SDS–PAGE analysis (a) and western blot analysis (b) of total extracellular proteins from *B. subtilis* 1A747 induced by maltose, harbouring those three strong promoters (lane 1) or not (lane 2). Bands of sublancin indicated by arrow presented in (a) were confirmed by western blotting (b). Marker lane, broad range protein marker (#P7702, New England Biolabs, USA).

Recombinant *B. subtilis* 1A747 strains were cultured in modified Medium A [2,24] at 37°C and induced at 12^th^ hour after fermentation start according to bacterial strain growth curve (Figure 3a). Meantime, the same culture system without maltose treatment served as a positive control and non–recombinant *B. subtilis* 1A747 strain with maltose treatment served as a negative control (Figure 3a). That the transcript amount of *sunA* and relative genes in recombinant strain induced by 5% maltose were 2 to 5 times higher than those of negative control when these strains reached the late logarithmic phase (Figure 3a and b) suggested that recombinant *B. subtilis* 1A747 strains have an obvious advantage in effective biosynthesis of mature sublancin (Figure 3c).

### Improved biosynthesis of mature sublancin

A maximum amount of 642 mg sublancin from 1 L culture supernatant of recombinant strain was achieved (Figure 3c), which is statistically significantly higher than those of 67 mg/L with maltose treatment (Figure 3c) and at most 60 mg/L without maltose treatment [2] both obtained from non–recombinant parental *B. subtilis* 1A747. Electrospray ionization mass spectrometry analysis showed that molecular weight of purified mature sublancin was 3877.642 KDa (Additional file 2), which is consistent with previous reports [2,5] and the result of tricine–SDS–PAGE analysis of 3.9 KDa (Figure 3d–a). Western blot analysis confirmed the result of tricine–SDS–PAGE analysis and showed that sublancin existed in culture supernatant (Figure 3d–b). The analysis of circular dichroism spectrum showed that purified mature sublancin in the liposome solution had a double–negative peak at 207 and 222 nm and thus displayed an α–helical conformation (Additional file 3) that is also in agreement with other study [25].

The above findings suggest the importance and advantages of this recombinant DNA technology in effective and efficient biosynthesis of mature sublancin. Recently, the synthesis of this type S–linked glycopeptide has become an interesting target through a complicated chemical [26] or semi–chemical [5] method, and several S–linked glycopeptides have been chemically synthesized [27-30]. However, both chemical and semi–chemical methods are suitable for small and linear glycopeptide synthesis but not for complicated glycopeptides like sublancin. The biosynthesis of S–linked glycopeptides with disulfide bridges such as sublancin using recombinant DNA technology has been rarely reported.

Several gene clusters such as those responsible for synthesizing mature nisin or subtilin in other microorganisms [31] are similar to that of sublancin, and also include the genes for precursor peptides and post–transcriptional modification enzymes. Their core peptides have different sequence identities but also contain abundant cysteine residues. In future, these specific post–transcriptionally modified antimicrobial peptides may become more common than currently appreciated. Therefore, it is assumed that more antimicrobial agents with post–transcriptional modification such as S–linked glycopeptides could be efficiently biosynthesized when to increase the possibilities of *in situ* utilizing vegetative or other appropriate promoters to adjust the transcriptional regulatory circuits of relative gene clusters.

### Determination of conditional stability

**Figure 4:**
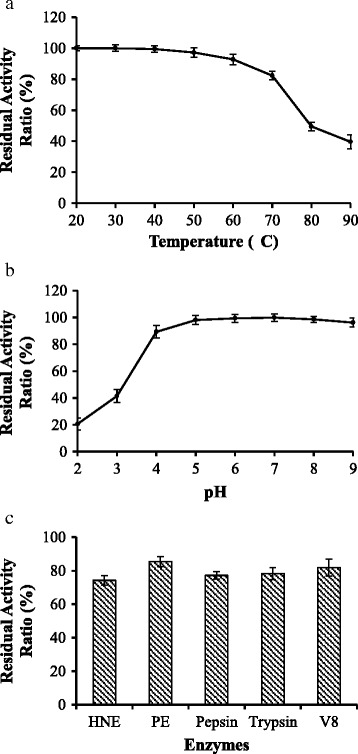
**Effects of different temperatures (a), pH (b), and enzymes (c) on sublancin.** The original reaction systems treated at 30°C and pH 7.0 were designated as the positive control, whereas the identical reaction systems without sublancin were used correspondingly as the negative controls. The residual activity ratios were calculated according to the description in the text.

As a potential alternative to conventional antibiotics in treating some bacterial–mediated inflammations like mastitis and gastroenteritis, the conditional stabilities of sublancin were evaluated using the simulated *in vivo* conditions i.e., specific pH, different temperatures and protease–rich environments. Our data show that temperatures ranging from 20°C to 70°C for 30 min can slightly affect the antimicrobial potency of sublancin (Figure 4a), while pH from 4.0 to 9.0 did not show significant effect on the antimicrobial activity (Figure 4b). The pH in body blood maintains at between 7.25 to 7.45 [32], whereas gastric juice, proximal small intestine, terminal ileum and cecum constantly maintain mean pH at 1.0 to 2.5, 6.6, 7.5, and 6.4, respectively [33]. Thus, these tissues besides stomach could provide optimum environments for potent biological activities of sublancin.

Proteolytic susceptibility of sublancin should also be considered in various applications such as in treating bacterial–mediated mastitis or bacterial–mediated gastroenteritis. Therefore, a number of bacterial proteases and gastrointestinal digestive enzymes, e.g., *P. aeruginosa* elastase and *S. aureus* V8 protease, pepsin and trypsin would certainly be encountered [34]. The results showed that sublancin maintained a majority of its activities when exposed to these proteases (Figure 4c). All experiments were performed *in vitro*; therefore *in vivo* studies should be conducted prior to clinical applications. Similar study about conditional stability of sublancin has not been previously documented. In contrast, a remarkable antimicrobial potency reduction of Bovicin HC5 was observed in *Streptococcus bovis* HC5 when exposed with proteinases, peptidases and heat [35], which can represent a common problem with regard to easy degradability of antimicrobial peptide.

Sugar linkages like glucose to cysteine can produce more stable products than conjugation to serine at high or low pH [36-38]. Mechanism of conditional stability of sublancin may be attributed to two disulfide bridges. Their coupling with glucose glycosylation probably provide the exceptional stability for sublancin by reducing the configurational entropy of unfolding; thus, conformational constraints are exerted, and conformational and biochemical stability are conferred on this agent [5].

### Activity of sublancin

Antibacterial activity and cytotoxicity of sublancin were also evaluated. Sublancin showed a potent antimicrobial activity towards nine bacterial strains including five typical and four drug–resistant strains, especially against *S. aureus* with MIC < 0.4 mg/L (Table 1). These findings are consistent with previously reported study [2] and is almost equal to that of nisin that is a common and typical antimicrobial peptide from *Lactococcus lactis,* and is widely used in clinical and food preservation applications [39], with MIC of 0.5 mg/L against *S. aureus*. In addition, sublancin exhibited an IC_50_ > 200 mg/L toward HT–29 cells, indicating its moderate cytotoxicity.

**Table 1:** **MICs of sublancin for different strains**

**Strains**	**MIC (mg/L)**
*E. faecalis* ATCC 29212	7.3 ± 0.35
gentamicin–resistant *E. faecalis*	6.8 ± 0.29
*S. aureus* ATCC 25923	0.6 ± 0.14
methicillin–resistant *S. aureus*	0.4 ± 0.21
*S. agalactiae* ATCC 27956	2.1 ± 0.35
erythromycin-resistant *S. agalactiae*	1.4 ± 0.24
*S. pyogenes* ATCC 19615	0.8 ± 0.22
erythromycin–resistant *S. pyogenes*	1.0 ± 0.36
*B. cereus* ATCC 10987	3.4 ± 0.21

The data derived from average values for three independent replicate experiments and almost identical triplicate sets of data.

An almost unaffected growth curve of recombinant *B. subtilis* 1A747 strain under high sublancin concentration was observed after the maltose induction (Figure 3a and c), hence confirming that the producer possessed the immunity against sublancin owning to the contribution of SunI generated simultaneously with sublancin [6].

## Conclusions

In our present study, we for the first time developed a recombinant *B. subtilis* 1A747 strain by displacing native promoters of genes responsible for mature sublancin biosynthesis with three distinctive promoters of P43, P_*glv*_ and P_*luxS*_, and the developed recombinant strain is capable of efficiently producing sublancin. The biosynthesized mature sublancin showed a rational molecular weight and conformation, displayed a potent and broad activity spectrum with a moderate cytotoxicity and illustrated a good conditional stability under the treatment with high temperatures, extreme pH and specific proteases. These findings are important for this recombinant strain in allowing for effective and efficient biosynthesis of sublancin. The promoter replacement provides an extra choice for high production of some other complicated peptide such as nisin and subtilin.

## Materials and methods

### Construction of mutant strains of *B. subtilis* 168

The pDM035 (Figure 2a) (kept in our laboratory), a shuttle vector able to replicate both in *E.coli* and *B. subtilis* and containing resistance genes against chloramphenicol and spectinomycin, was double digested with *Xho*I and *Xba*I. The fusion fragment of P43–*sunI*–P_*glv*_–*sunA*–P_*luxS*_–two homologous arms with *Xho*I and *Sac*I (Figure 2b) was synthesized at AuGCT Co. Ltd. (Beijing, China). The fragment was inserted into vector pDM035 by displacing β–galactosidase gene to generate pDM036 (Figure 2c). The resultant pDM036 was transferred into *Escherichia coli*, and the positive clones were selected using 5 μg/mL chloramphenicol. *B. subtilis* 1A747 [SPβc, prototroph, derivative of *B. subtilis* 168 (trpC2)] (*Bacillus* Genetic Stock Center, USA) competent cells were transformed with linearized pDM036 digested with *Bam*HI using the electroporation approach [40].

The positive–recombinant *B. subtilis* cells were selected from LB agar with 5 μg/mL chloramphenicol and 50 μg/mL spectinomycin resistances. These strains were cultured in 50 mL modified Medium A [2,24] in 250 mL shake flask with agitation speed of 225 rpm at 37°C. The culture mixture was not supplied with 5% maltose until the late logarithmic phase of strains [41]. After another 24 h culture, the fermentation broth was harvested and centrifuged at 12,000 × *g* for 15 min at 4°C and the supernatants were collected. *B. subtilis* 1A747 strains were used as control that was performed as recombinant *B. subtilis* was done. Crude sublancin concentrations in fermentation supernatant were measured by HPLC using the method described below.

### Isolation of total RNA and Real–time PCR

The cultures were collected once every 3 h from 3 h until 48 h after fermentation. Total bacterial RNAs were isolated by a SV total RNA isolation kit (#Z3100; Promega, USA). Extracted total RNA was reverse–transcribed into cDNA chain by a Reverse Transcription System Kit (#A3500; Promega, USA). Real–time PCR was carried out using Real time PCR Kit (#DRR041S; TaKaRa, Japan). The genes of *sunI*, *sunA*, *sunT*, *bdbA*, *sunS* and *bdbB* were amplified by primers as shown in Table 2. 16 s rDNA of *B. subtilis* 168 was amplified as control using 16 s–up/16 s–down primers. PCR protocols were as follows: 2 min at 50°C and 10 min at 95°C, followed by 35 cycles consisting of 42 s at 95°C, 60 s at the annealing temperatures shown in Table 2, and 30 s at 72°C. Reactions were performed in IQ5 Real–time PCR detection system (Bio–RAD, USA).

**Table 2:** **Primers and annealing temperatures used in real–time PCR**

**Gene**	**Primer**	**Sequence**	**Annealing temperature (°C)**
*sunI*	sunI–up	AAGAGTCAGACAAGTATGGAGTT	48
	sunI–down	TTAAATGGAGCTCAACAATTTA	
*sunA*	sunA–up	GAACTGGAAAATCAAAAAGGT	49
	sunA–down	CAAAACTGCCGGTAATTCT	
*sunT*	sunT–up	GGGGATAAGGAAGGCTATAG	50
	sunT–down	TAATGTCCATATTCCTCCCC	
*bdbA*	bdbA–up	GCAGCAGCCATTAGTATTTTC	51
	bdbA–down	CAAGGAGGACAACTTGTCTCA	
*sunS*	sunS–up	GGCTATGCCGATTCTTTATT	50
	sunS–down	CCGCATGTTATTGTAGGAGTA	
*bdbB*	bdbB–up	CCATGTGTTCTATGTTGGTATC	49
	bdbB–down	CCAATTTCACATACGACACTT	

### Western blotting

The culture supernatants at 36 h were collected at 12000 g for 10 min, mixed with 4 × Laemmli loading buffer (3:1) and heated in boiling water for 5 min. Subsequently, the tricine sodium dodecyl sulfate polyacrylamide gel electrophoresis (tricine–SDS–PAGE) [42] analysis was performed using 10% gel (Figure 3d–a) and then electro–transferred to PVDF membrane (Millipore, USA) for protein immunoblot analysis (Figure 3d–b). The preparation and purification of mouse anti–sublancin monoclonal antibody was performed by Cwbiotech (Beijing, China). After incubation with HRP–conjugate secondary antibody, bands were visualized by chemiluminescence using a ChemiDoc XRS imaging system and analysis software Quantity One (Bio–Rad, USA).

### Purification of sublancin

Isolation and purification of sublancin were performed as described previously [2] with slight modification. The harvested supernatant was placed in 1 M NaCl and then subjected to a hydrophobic interaction chromatography using 25 mL Toyo pearl Butyl–650 column (Tosoh, Tokyo, Japan), equilibrated with 1 M NaCl and 50 mM NaAc (pH 4.0). The unbound proteins were washed with loading buffer, and 50 mM NaAc (pH 4.0) was used to elute sublancin. The elution was placed in 0.1% TFA and subjected to HPLC using a semi–preparative Zorbax 300SB–C8 column (250 mm × 9.4 mm, 5 μm particle size, 300 Å pore size; Agilent, Englewood, CO), equilibrated in 0.1% (v/v) TFA and 10% acetonitrile. The elution was subsequently developed with a linear 0% to 60% acetonitrile gradient at a flow rate of 1.0 mL/min. Fractions from different retention times were tested for antimicrobial activity. Active fractions were collected and then subjected to HPLC using an analytical Zorbax 300SB–C8 column (150 mm × 4.6 mm, 5 μm particle size, 300 Å pore size; Agilent, Englewood, CO) under the same conditions as used in the first step. The absorbances at 214, 254 and 280 nm were monitored and *S. aureus* was designated as indicator strain in antimicrobial activity assays. The concentration of purified sublancin was determined by UV spectrophotometry [43,44]. The molecular weights of sublancin were obtained by electrospray ionization mass spectrometry (Agilent, USA; Figure 4). The pooled solution of sublancin was freeze–dried in a vacuum freeze dryer (SIM International Group Co., Ltd., USA) at −80°C for further experiment.

### Secondary structure analysis

Using a 1 mm path–length quartz cuvette, the second structure of sublancin at 50 mg/L in Palmitoyl–oleoyl–phosphatidylglycerol (POPG) liposome solution was detected using a Jasco 810 spectropolarimeter (Jasco Corporation, Japan) at room temperature, within the range of 190 nm to 250 nm at 10 nm/min, as described previously [45]. The liposome solution without sublancin was used as a reference. POPG liposomes (Sigma, USA) were prepared with the following slight modification: a specific amount of POPG was dissolved into 10 mM PBS (pH 7.4) to prepare the stock solution of 100 μM POPG.

### Antimicrobial activity

*B. cereus* ATCC 10987, *Enterococcus faecalis* ATCC 29212, *S. aureus* ATCC 25923, *S. agalactiae* ATCC 27956, and *S. pyogenes* ATCC19615 were obtained from ATCC (Rockville, MD, USA). Erythromycin–resistant *S. agalactiae*, erythromycin–resistant *S. pyogenes*, gentamicin–resistant *E. faecalis* and methicillin–resistant *S. aureus* were kept in our laboratory. MIC assay was performed depending on a microtiter broth dilution method, as described previously [46], with slight modification. A packed volume with DEAE–Sephacel (Sigma–Aldrich, Schnelldorf, Germany) was equilibrated with deionized water, 2 M NaCl, 0.1 M NaOH, 70% ethanol, and 10 mM Tris buffer (pH 7.4). Approximately 100 mL of LB broth in the same Tris buffer was subjected to the treated column to prepare a refined medium, and then sterilized using 0.22 μL of the membrane filter. In addition, the peptide sample was dissolved into Tris buffer to prepare 10–fold serial dilutions. After overnight culture, the tested strains were rinsed with Tris buffer and diluted to 5 × 10^5^ CFU/mL in refined LB medium. Aliquots of 90 μL of bacteria solution were added into the wells of a 96–well microtiter plate. Subsequently, 10 μL of the serial 10–fold dilutions were placed into corresponding wells and produced a serial working concentration of 64, 32, 16, 8, 4, 2, 1, 0.5, 0.25, 0.12 and 0.06 mg/L. The mixtures were incubated at 37°C for 21 h, and then measured as described previously [47]. A negative control was also prepared using same reaction system without sublancin. These experiments were repeated three times (hereinafter the same). MIC was defined as the lowest concentration of an antimicrobial agent required to inhibit 90% of microorganism growth after overnight incubation.

### HT–29 lytic activity

The human colorectal adenocarcinoma cell line HT–29 were obtained from the ATCC and cultured in RPMI–1640 medium (Invitrogen, USA) supplemented with 0.2 g/L streptomycin, 0.1 g/L penicillin, 10% heat–inactivated fetal calf serum (FCS, Germany). Cells were maintained at 37°C in 5% CO_2_. For assays, the HT–29 cells were first starved for 24 h in serum–free medium, and then were seeded in a 24–well plate (Nunc, Germany) at 1 × 10^5^ cells/well. At subconfluency, medium was replaced, and the cells were incubated with the serial sublancin dilutions of 200, 100, 50, 25, 12, 6, and 3 mg/L in a volume of 100 μl for 24 h. Cell viability was assessed in exposed cultures by using a colorimetric 3–(4,5–dimethylthiazol–2–yl)–2,5 diphenyltetrazoliumthiazolyl blue assay (MTT, Roche Diagnostics, Germany). The reaction samples were detected at 570 nm with a microtiter ELISA reader (Epoch^TM^, BioTek–® instruments, Inc., USA).

### Assessment of stability

The effect of different factors including enzymes, pH and temperatures on sublancin stability was evaluated. The evaluation was performed in accordance to the method described in the antimicrobial activity section with slight modification. Aliquots of 5 mg/L sublancin in PBS (pH 7.4) were treated under different protease [HNE (Innovative Research, Novi, MI), PE (Innovative Research), pepsin (Sigma), V8 (BioCol GmbH), and trypsin (Sigma Chemical Co., St. Louis, MO)] at a substrate: protease molar ratio of 300:1 at 37°C, gradient pH values (pH 2.0, 3.0, 4.0, 5.0, 6.0, 7.0, 8.0, and 9.0) at 37°C and gradient temperatures (20, 30, 40, 50, 60, 70, 80 and 90°C), all treatments were performed for 30 min. After the treatment, the indicator bacteria were mixed with 100 μL treated peptide solution. Approximately 5 × 10^5^ CFU/mL solution was obtained and then incubated at 37°C for 21 h. At the same time, a similar reaction system at 30°C (pH 7.0) was set as positive control; the same systems without sublancin were correspondingly used as negative controls. *S. aureus* was used as indicator strain. The effect of these factors on sublancin stability was evaluated through residual activity using the following formula:

$$ \mathrm{residual}\kern0.5em \mathrm{activity}\kern0.5em \mathrm{ratio}\kern0.5em \left(\%\right)=\left({A}_1\hbox{--} A\right)/\left({A}_1\hbox{--} {A}_2\right)\times 100\% $$

where *A*, *A*_1_, and *A*_2_ represent the absorbance of the different factors, negative controls and positive control, respectively.

### Statistical analysis

The experiments were repeated for three times, and the mean values were expressed as mean ± standard deviation.

## Additional files

Additional file 1. Regulation net of sublancin biosynthesis. Additional file 2. Electrospray ionization mass spectrometry analysis for mature sublancin. Additional file 3. Circular dichroism spectrum of sublancin in liposome solution.

## Abbreviations

ECF σ factors: Extra cytoplasmic function σ factors
LB: Lysogeny broth
HPLC: High performance liquid chromatography
MTT: 3–(4, 5–dimethylthiazol–2–yl)–2, 5 diphenyltetrazoliumthiazolyl blue
TFA: Trifluoroacetic acid
POPG: Palmitoyl–oleoyl–phosphatidylglycerol
CFU: Colony–forming units
ATCC: American Type Culture Collection
MIC: Minimum inhibitory concentration
HNE: Human neutrophil elastase
PEY: *P. aeruginosa* elastase
V8:*S. aureus*: V8 protease
