# Supplementary material for: Improved production of sublancin via introduction of three characteristic promoters into operon clusters responsible for this novel distinct glycopeptide biosynthesis
Source: Microb Cell Fact. 2015 Feb 12;14:17. doi: 10.1186/s12934-015-0201-0 (PMC4336743; doi:10.1186/s12934-015-0201-0)
Supplement: Additional file 1. — Regulation net of sublancin biosynthesis. [file 12934_2015_201_MOESM1_ESM.docx]

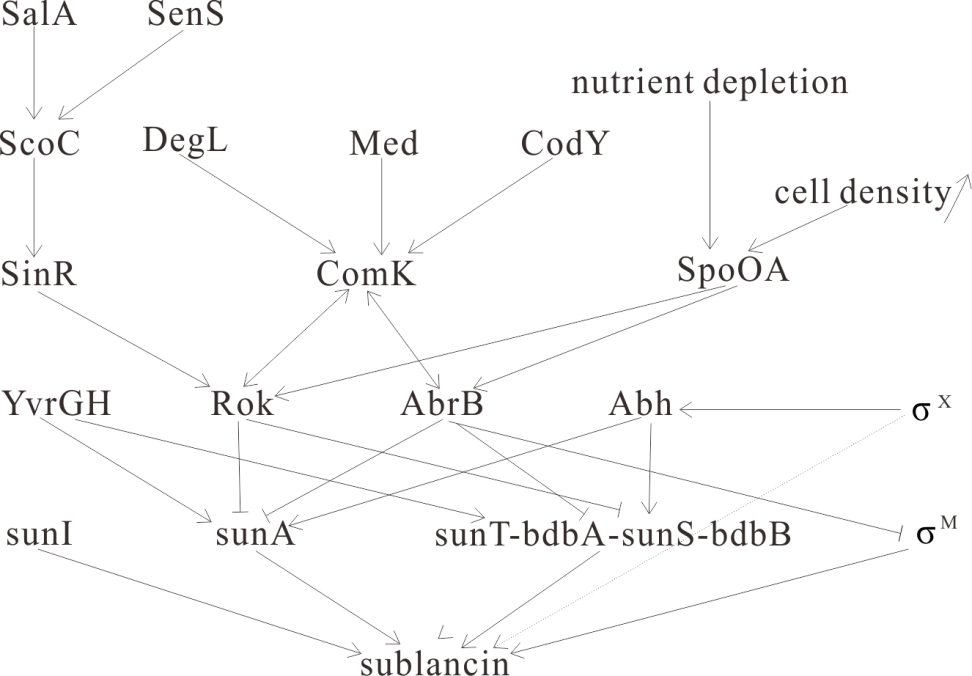


Additional file 1 Regulation net of sublancin biosynthesis. Sublancin biosynthesis is controlled under a complex regulatory network and involves a minimum of five transcriptional regulators (Abh, AbrB, Rok, YvrG, and YvrH) and a minimum of two ECF σ factors (σ^M^ and σ^X^). "→" indicates a positive regulation and “┴” indicates a negative regulation.
