# Supplementary figures and images for: Improved production of sublancin via introduction of three characteristic promoters into operon clusters responsible for this novel distinct glycopeptide biosynthesis
Source: Microb Cell Fact. 2015 Feb 12;14:17. doi: 10.1186/s12934-015-0201-0 (PMC4336743; doi:10.1186/s12934-015-0201-0)

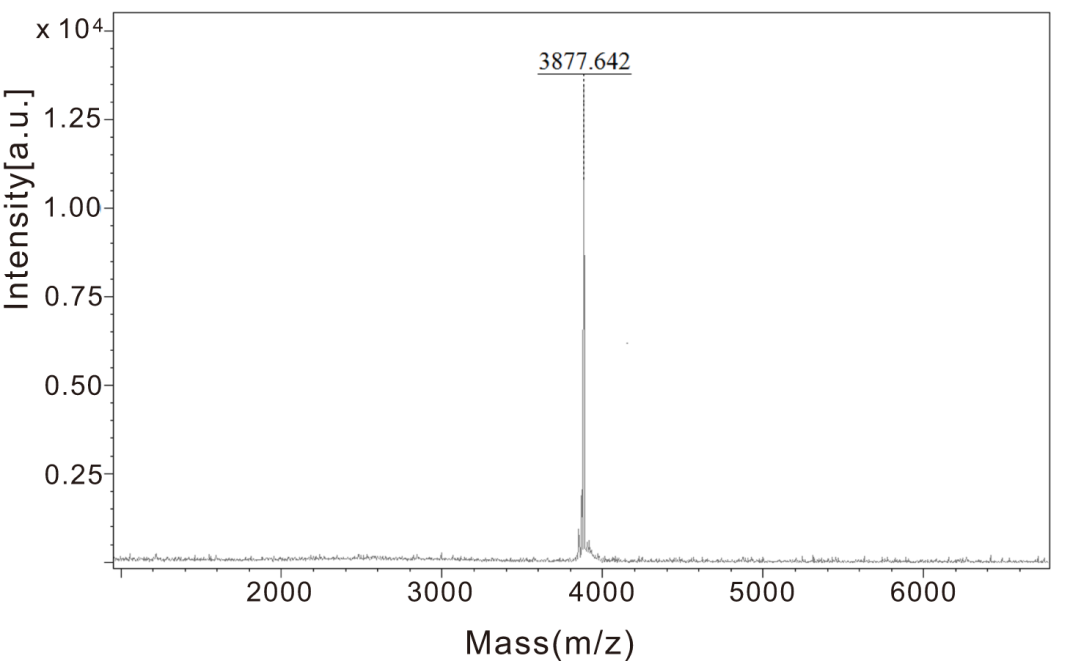


Additional file 2 Electrospray ionization mass spectrometry analysis for mature sublancin.

Supplement: Additional file 2. — Electrospray ionization mass spectrometry analysis for mature sublancin. [file 12934_2015_201_MOESM2_ESM.docx]
