# Supplementary material for: Improved production of sublancin via introduction of three characteristic promoters into operon clusters responsible for this novel distinct glycopeptide biosynthesis
Source: Microb Cell Fact. 2015 Feb 12;14:17. doi: 10.1186/s12934-015-0201-0 (PMC4336743; doi:10.1186/s12934-015-0201-0)
Supplement: Additional file 3. — Circular dichroism spectrum of sublancin in liposome solution. [file 12934_2015_201_MOESM3_ESM.docx]

Additional file 3. Circular dichroism spectrum of sublancin in liposome solution.
